# *In-vitro* fermentation characteristics and methane reduction potential of mustard cake (*Brassica juncea* L.)

**DOI:** 10.14202/vetworld.2016.1141-1146

**Published:** 2016-10-26

**Authors:** S. M. Durge, M. K. Tripathi, N. Dutta

**Affiliations:** 1 Division of Nutrition, Feed Resource and Product Technology, Central Institute for Research on Goats, Mathura - 281 122, Uttar Pradesh, India; 2 Division of Animal Nutrition, Indian Veterinary Research Institute, Bareilly - 243 122, Uttar Pradesh, India; 3 Department of Instructional Livestock Farm Complex, College of Veterinary and Animal Sciences, Udgir, Latur - 413 517, Maharashtra, India

**Keywords:** *Brassica*, glucosinolate, mustard cake, methane, rumen fermentation

## Abstract

**Aim:**

To assess the effect of mustard cake (*Brassica juncea* L.) levels in concentrate mixtures and in composite feed mixtures (CFMs) on *in-vitro* fermentation characteristics and methane production.

**Materials and Methods:**

Five concentrate mixtures were prepared with containing 30% oil cake, where linseed cake was replaced by mustard cake at the rate of 0%, 7.5%, 15.0%, 22.5%, and 30% in concentrate mixture. Mustard cake contained glucosinolate 72.58 µmol/g oil free dry matter (DM) and contents in diet were 0, 5.4, 10.9, 16.3, and 21.8 µmol/g of concentrate mixture, respectively. Concentrate mixture containing 15.0% mustard cake was found to produced minimum methane which was then used for the preparation of CFM containing 0%, 25%, 50%, and 75% levels with gram straw.

**Result:**

Increased levels of mustard cake in concentrate mixtures had a linear decrease (p<0.05) in the total gas production, and the 15% inclusion showed lowest methane concentration (quadratic, p<0.01). The degradability of DM and organic matter (OM) of concentrate mixtures did not change, however, pH and NH_3_-N concentrations of the fermentation medium showed linear (p<0.05) reductions with increased mustard cake levels. Increased levels of 15% mustard cake containing concentrate mixture in CFMs exhibited a trend (p=0.052) of increased gas production, whereas methane concentration in total gas, methane produced and degradability of DM and OM were also displayed a linear increase (p<0.05). However, the pH, NH_3_-N, and total volatile fatty acid levels decreased linearly (p<0.05) with increased levels of concentrate in CFMs.

**Conclusion:**

Reduction in methane production was evidenced with the inclusion of mustard cake in concentrate mixture at 15% level, and the CFMs with 25% concentrate, which contained 15% mustard cake, exhibited an improved fermentation and reduced methane production.

## Introduction

Methane emitted by ruminants contributes sizably to the global methane emissions apart from representing a loss of feed energy, which can be used for productive purposes. Methane produced by ruminants contributes 20% of the total emissions through agricultural activities, in which each gram of methane production causes 55.65 kJ feed energy loss [1]. Ruminal methane emission is a synergy process between hydrogen producing and hydrogen utilizing resident microbes. The magnitude of methane emissions from rumen is affected by several factors, where diet and feeding, in particular, have great influence. Dietary modifications have been recommended as the viable strategies for decreasing methane production in rumen ecosystem [2]. Dietary strategies, which do not have adverse effect on rumen ecology, could be exploited to reduce methane emission from ruminants. The inclusion of products and by-products of *Brassica* spp. were recommended in ruminant diets for methane mitigation [3]. Mustard cake, a by-product of oil extracting industries, is a cheap and easily available protein source for animal feeding in India [4]; this could be utilized in ruminant feeding as a protein supplement to reduced ruminal methane production.

The feeding cost accounts more than 70% of the total production cost of livestock, and therefore, the economization of the cost of feeding is a challenge for animal nutritionists. Protein supplements are the most costly feed ingredients used in animal feed. Alternate feed resources are added to the feed chain to increase the availability of feed resources and reduce the cost of feeding. Groundnut cake, soybean meal, linseed, and til cake are conventional protein supplements used in goat feeding. However, these are very costly, and availability for ruminant feeding is also limited. Mustard cake is widely available at cheaper prices but its utilization in goat feeding is limited because of its bitter taste and glucosinolate content [5]. *Brassica* forage and oil meals have showed a role in reducing methane production in the rumen [3,6]. *Brassica* forages may be a viable option for methane mitigation in pastoral animal production system [3], whereas *Brassica* oil meals can be adopted in pen feeding or supplementary feeding based livestock production for reducing methane production.

Therefore, keeping in view the potential availability, low cost and effect on rumen methane mitigation of *Brassica* products, the aim of this study was to assess the effect of mustard cake (*Brassica juncea* L.) levels in concentrate mixtures and in composite diets on *in-vitro* fermentation characteristics and methane reduction.

## Materials and Methods

### Ethical approval

The experimental protocol was duly cleared by the Institute Animal Ethic Committee.

### Animals, housing and feeding management

Four adult Barbari male goats penned in a well-ventilated enclosure were fed on a composite diet comprising roughage (gram straw) to concentrate (linseed and mustard cakes 15.0% of each as protein supplements, barley 42.0, wheat bran 25.0, mineral mixture 2.0, and common salt 1.0%) in the ratio of 70:30. Three animals were used as donor of microbial inoculums during *in-vitro* study, and the nutrient requirement of donor animals was met as per recommendations [7].

### Feed formulations for *in-vitro* fermentation

#### Experiment 1

Five concentrate mixtures were formulated with mustard cake levels of 0 (MC-0), 7.5 (MC-7.5), 15.0 (MC-15.0), 22.5 (MC-22.5), and 30% (MC-30.0) replacing (w/w) linseed cake at 0%, 25%, 50%, 75%, and 100%, respectively (Table-1). The fermentation gas and methane production characteristics and the digestibility were determined. The concentrate mixture, which produced the lowest methane, was used in subsequent experiments.

**Table-1:** Composition of concentrated mixture.

Ingredients	Concentrated mixtures^a^				

	MC-0	MC-7.5	MC-15	MC-22.5	MC-30
Ingredient composition (g/100 g)					
Barley	42	42	42	42	42
Wheat bran	25	25	25	25	25
Linseed cake	30	22.5	15	7	0
Mustard cake	0	7.5	15	22.5	30
Mineral mixture^b^	2	2	2	2	2
Common salt	1	1	1	1	1
Chemical composition (g/100 g DM)					
OM	92.73	92.86	92.94	93.03	93.51
Crude protein	20.10	19.97	19.86	19.97	20.05
Glucosinolate (µmol/g)	0	5.44	10.89	16.33	21.77

a Mustard cake level in concentrate feed (g/100 g feed),

b Contained Ca 240, P 90, Mn 1.2, iron 6, Cu 1, Co 0.2, sodium chloride 300, iodine 1, and fluorine<0.3 g/kg. OM=Organic matter, DM=Dry matter

#### Experiment 2

The concentrate mixture (MC-15.0) was incorporated at 0%, 25%, 50%, and 75% levels in gram straw to formulate composite feed mixtures (CFM) of varying roughage and concentrate ratio, these CFMs have simulated the different feeding systems of goats.

### *In-vitro* fermentation

The *in-vitro* fermentation studies were carried out in 100 ml glass syringes. In brief, the concentrate mixtures and CFMs were ground to pass 1 mm screen. A 200 mg homogenized sample was placed at the bottom of glass syringe, mixed with 30 ml microbial inoculums and incubated at 39°C for 24 h. The microbial inoculums used with slight modifications [8], inoculums contained distilled water 365 ml, buffer solution 183 ml, solution of macrominerals 183 ml and microminerals 100 µl, strained rumen fluid 330 ml, resazurin 0.01 mg and reducing solution 38.8 ml. The carbon dioxide was fluxed appropriately to maintain anaerobic conditions during fermentation. Each sample was incubated in triplicate with inoculums from three individual animals. Total gas production was measured by piston displacement method for 24 h. The volume of gas formed was converted to mmol assuming 1 mol of gas was equivalent to 22.4 L of gas under the atmospheric pressure and temperature conditions of measurements. The methane content of the gas was analyzed, and fermentation metabolites were assessed in fermentation medium.

### Methane estimation

The methane in fermented gas mixture was estimated using a gas chromatograph (GC) (PerkinElmerClarus-580, Singapore). A dual channel, dual column, microprocessor based GC was equipped with ECD and FDI detector and control module for temperature controls. Stainless steel column, which was suitable for methane analysis was used. The airtight sterilized syringes were used to withdraw the gas from the *in-vitro* syringes at the rubber outlet, which was injected in the GC and area of the graph plotted by output system was recorded for methane estimation.

### Chemical analysis

The dry matter (DM) was determined by drying at 70°C until constant weight. Organic matter (OM) was estimated by ashing the dried samples at 450°C for 4 h as per AOAC [9]. The protein contents were determined by Kjeldahl technique (N×6.25) [9]. After 24 h incubation, pH of the fermentation medium was measured by the portable electronic pH meter (PCSTestr 35, Eutech Oakton Instruments, IL 60061, USA). Ammonia nitrogen was determined by the method of Weatherburn [10], and the total volatile fatty acids (TVFA) were estimated following the procedure of Barnett and Reid [11]. The glucosinolate content in mustard cake, concentrate mixtures, and CFMs was estimated [12] following Sephadex (DEAE-Sephadex A-25)-pyridine-acetate column procedure for glucosinolate purification [13].

### Statistical analysis

The observations of gas and methane production, DM and OM digestibility, and fermentation characteristics were analyzed for statistically significance by analysis of variance procedure using a general linear mathematical model as: Y_ijk_ = µ + T_i_ + e_ij_, Where: Y_ijk_ = Observation mean; µ = General mean, T_i_ = Effect of i^th^ treatment (i = 1, 4 or 5), e_ij_ = Random error. The above model was also tested for level of significance using linear and quadratic effects against MC-0 in CFMs (SPSS base 16).

## Results

### Experiment 1: Fermentation and methane production of concentrate mixtures

Increased levels of mustard cake in concentrates reduced (p<0.05) total gas production, which ranged from 178 to 222 ml/g DM among different concentrates (Table-2). The concentration of methane in total gas ranged from 10.64% to 14.47%, which was significantly (p<0.05) different among five concentrates. The concentrate mixture (MC-15) had the lowest methane concentration in total gas and showed a quadratic (p<0.01) decrease in methane production. In spite of different methane concentrations in the total gas, methane production in terms of L/kg DM and g/kg DM were similar among five concentrates. The protein content of mustard and linseed cake was identical. Mustard cake had glucosinolate 72.58 µmol/g oil free DM, which contributed glucosinolate 5.4, 10.9, 16.3, and 21.8 µmol/g concentrate, respectively, in MC-7.5, MC-15.0, MC-22.5, and MC-30 (Table-1). Inclusion of MC up to 30% in concentrate did not change the digestibility of DM and OM. However, the pH of the fermentation medium was reduced linearly (p<0.05) with increased levels of mustard cake in concentrates. Mustard cake levels had a significant (p<0.05) influence on NH_3_-N concentration of fermentation medium. The MC-0 concentrate mixture had the highest (10.7 mg/L) NH_3_-N, and the lowest (4.4 mg/L) NH_3_-N was in MC-22.5 concentrate mixture (Table-2). Increased MC levels showed a linear (p<0.001) decreased NH_3_-N concentrations of the fermentation mediums. Although MC inclusion levels did not show a significant change in TVFA concentrations, however a trend of decreased (p=0.057) TVFA in fermentation medium was observed with increased MC levels in concentrates. The *in-vitro* fermentation of concentrates demonstrated that the MC at 15% level had the minimum methane concentration in fermentation gas and this level of MC also produced the lowest quantity of methane on each unit of concentrate incubated.

**Table-2:** *In-vitro* fermentation gas and fermentation characteristics of the concentrate mixtures having graded levels of mustard cake (means of three observations).

Parameters	Concentrate mixture^a^					SEM	Significance		
	
	MC-0	MC-7.5	MC-15.0	MC-22.5	MC-30.0		Treat	Linear	Quadratic
Fermentation characteristics									
pH	6.89	6.84	6.85	6.79	6.80	0.012	0.071	0.011	0.537
NH_3_-N (mg/L)	10.78	8.97	5.95	4.45	4.75	0.779	0.007	<0.001	0.186
TVFA (mmole/L)	23.3	21.3	18.6	11.6	11.3	2.294	0.355	0.057	0.958
Gas production characteristics									
Gas (ml/g DM)	195.4	222.0	199.5	177.9	179.5	5.834	0.077	0.045	0.238
Methane (%)	14.47	11.76	10.64	12.44	14.07	0.435	0.003	0.946	<0.001
Methane (L/kg)	37.14	37.20	31.07	33.82	38.44	1.320	0.419	0.935	0.131
Methane (g/kg)	26.59	26.64	22.25	24.22	27.53	0.944	0.419	0.935	0.131
Digestibility									
DM	68.27	59.83	64.53	50.72	69.25	2.900	0.251	0.714	0.146
OM	65.32	57.98	61.84	48.58	67.64	2.943	0.283	0.813	0.154

a Mustard cake level in concentrate feed (g/100 g feed). OM=Organic matter, DM=Dry matter, TVFA=Total volatile fatty acid, SEM=Standard error of mean

### Experiment 2: Fermentation and methane production of CFMs

The total gas production among four CFMs exhibited a trend (p<0.052) of increased gas production with increased levels of concentrate mixture. However, increased concentrate levels in CFMs revealed linearly (p<0.001) increase (L/kg or g/kg) in methane production up to 50% concentrate level (Table-3). Digestibility of DM and OM of CFMs increased (p<0.05) with increased levels of concentrate in CFMs. Similarly, efficiency of methane production (ml methane/g digestible OM) was reduced (p<0.001) with increased concentrate levels in CFMs. The CFM with 25% concentrate (mustard cake 15 g/100 g) showed the lowest methane production and thus had the higher methane reduction efficiency. Increased level of concentrates in CFMs showed a linear (p<0.01) decrease in the pH values of the fermentation medium, while CFMs with 25% concentrates revealed a quadratic (p<0.001) decrease in the pH value. Moreover, increased concentrate levels in CFMs revealed a linear decrease in NH_3_-N concentration (p<0.001) and TVFA levels (p<0.05) in the fermentation medium. However, no significant differences were in NH_3_-N concentrations among CFMs with 0%, 25%, and 50% concentrates. The TVFA levels of fermentation mediums did not demonstrate a definite trend.

**Table-3:** *In-vitro* fermentation characteristics of gram straw based substrate with varying R:C (mean of three observations).

Parameters	Complete feed mixture*				SEM	Significance		
	
	C-0	C-25	C-50	C-75		Treatment	Linear	Quadratic
Fermentation characteristics								
pH	7.18	6.81	6.62	6.61	0.070	0.090	<0.001	<0.001
NH_3_-N (mg/L)	12.59	14.10	8.37	3.39	1.413	<0.001	<0.001	0.067
TVFA (mmole/L)	26.0	30.0	19.3	12.0	2.424	0.012	0.024	0.593
Gas production characteristics								
Gas (ml/gDM)	86.2	132.0	117.9	134.5	7.974	0.091	0.052	0.262
Methane (%)	11.3	10.5	15.1	12.7	0.572	<0.001	<0.001	0.162
Methane (L/kg)	20.39	23.65	32.01	29.17	1.476	<0.001	<0.001	0.043
Methane (g/kg)	14.60	16.93	22.92	20.89	1.055	<0.001	<0.001	0.043
Digestibility								
DM	28.90	51.51	48.70	51.29	3.304	0.010	0.016	0.134
OM	26.43	48.30	47.60	46.77	3.271	0.032	0.038	0.204

* Level of concentrate (mustard cake 15 g/100 g) (g/100 g) in gram straw based CFM. CFM=Composite feed mixture, OM=Organic matter, DM=Dry matter, TVFA=Total volatile fatty acid, SEM=Standard error of mean

## Discussion

Decreased total gas production with increased mustard cake levels have corroborated favorably [14] and reported a low level of gas production in various rapeseed-mustard cakes with different levels of glucosinolates. However, an overall higher gas production in this study was due to higher fermentability of concentrate feeds in comparison to the fermentability of oil cakes alone. Although rapeseed-mustard cake contains good amount and quality of protein, glucosinolate content may limit its use in animal feeding. The glucosinolates themselves are biologically inactive molecules; however, glucosinolate degradation products are biologically active and known for their diversified biological effects. Negative effects of glucosinolate on animals are relative to its concentration in feed resource [5]. This study did not observe any adverse effect of mustard cake glucosinolate on DM digestibility, OM digestibility, and TVFA production because of low content in total diet [5]. Reduced levels of NH_3_-N together with TVFA in rumen fluid were indicative of improved microbial biomass synthesis [15], which in turn increased total gas production. There are three general approaches to mitigate methane emissions from ruminants. These include manipulation of rumen microbes, selection of animals (which naturally emit less methane) and nutritional management. Within nutritional interventions concentrates, oils, ionophores, nitrate, and sulfate have been examined and some of them are effective [16,17]. *Brassica* feeding in animals as a methane mitigant have been recommended [3]. The results of this study have indicated that concentrates with 15% mustard cake has a decreased rumen methane production. The varying levels of glucosinolate were not determined in this study. Increased methane production with higher level of concentrates in CFM might have accompanied by improved fermentation of feeds as *Brassica* products were more rapidly fermented [3] than the linseed cake/products, and mustard cake contained more proportion of rumen degradable proteins [18]. The product and by-products of *Brassica* (*Brassica oleracea* [Kale], *Brassica campestris* [turnip], *Brassica napus* [rape/swede], etc.) contained secondary plant metabolites including polyphenol oxidase, S-methyl cysteine sulfoxide, and glucosinolate [5], which might have altered gut bacterial communities particularly by the inclusion of glucosinolate-containing products in the diet [19]. Although there is no reported study on effects of any of these secondary plant metabolites on rumen methanogens, however a decreased methane production by 23% and 25%, respectively, with rape and swede compared to ryegrass was reported in a pastoral based sheep production system [3]. In this study, methane yield increased with increased digestibility of CFM which was accompanied by higher levels of concentrates, whereas efficiency of methane production (ml methane/g digestible OM) was decreased. Methanogenesis and methane production have a negative relationship with OM digestibility in sheep [3], where rape and swede grazing reduced methane emission by 25-43%. Contrary to these, earlier studies [19,20] did not find any relationship between digestibility and methane emissions. Although negative relationships between methane yield and digestibility, this phenomenon has suggested that the other factors have an overriding effect on methanogenesis in animals fed with *Brassica* feeds [3]. In this study, methane productions were not statistically different among the levels of mustard cake in concentrates; however, the methane production was gradually increased in concentrate mixtures with mustard cake levels of 22.5% and 30%. The increased methane production in concentrate with 22.5% and 30% mustard cake levels could not be explained with present set of observations. Reduced NH_3_-N and TVFA have indicated a promoted microbial synthesis [15], which increased total gas production but methane production was not changed across the levels of mustard cakes. This might be due to no effect of mustard cake on methanogenic microorganisms. However, methane production in *in-vitro* system might have differed from *in-vivo* system in animal studies. Moreover, pattern of methane yield changed with substrate quality. Therefore, further studies are required for delineating the metabolic pathways that are involved in methane reduction, which is associated with mustard/*Brassica* product feeding. Linear decrease in pH of fermentation medium showed that the mustard cake inclusion have improved the soluble carbohydrate levels. However, NH_3_-N decreased with increased levels of mustard cake in spite of higher degradable protein content in mustard cake than in linseed cake. Decreased NH_3_-N along with reduced VFA is known for improved microbial protein synthesis in rumen. The rumen microbes utilize NH_3_ as nitrogen source and VFA as source of energy for their cell growth. Improved microbial protein synthesis with increased mustard cake levels have also been reported [6]. These findings are also in agreement with the observations, where no adverse effect of rapeseed meal feeding on rumen fermentation was reported [18] irrespective of their glucosinolate content. The increased production of propionate was reported [6] with the addition of rapeseed meal or rapeseed extract as source of glucosinolate, which reduced pH of fermentation medium, as more glucose was released from the hydrolysis of glucosinolate [5]. It is well established that increased levels of concentrates in composite diets have improved fermentability and fermentation metabolites level. In this study, no significant differences were revealed in total gas productions in CFMs with the 25-75% concentrate levels. Whereas, pH, NH_3_-N, and VFA decreased linearly with increased concentrate levels. The decline in rumen pH was associated with slow ammonia absorption in rumen and the small changes in rumen pH can have a marked influence on ammonia absorption from the rumen, through its effect on unionized ammonia concentrations have also been reported [21,22]. Results of the study revealed that rumen ammonia was either rapidly absorbed or incorporated in the microbial mass, thus did not corroborate with total-N-concentration in the rumen fluid.

## Conclusions

The concentrate mixtures with 15% mustard cake have contributed 10.9 µmol glucosinolate/g DM, which resulted in decreased methane production without effecting fermentation metabolites. Inclusion of such concentrates at 25% level in CFMs (R: C; 75:25) improved fermentation and reduced methane production efficiency (ml/g digestible OM). Therefore, mustard cake could be used in ruminant feeding as a methane mitigant and could offer an economic option in feed formulation.

### Authors’ Contributions

SMD: Collection of ingredient, preparation of concentrate mixtures and composite feed mixtures, collection of rumen liquor, conduction of experiment, analysis of parameter, daily data recording and analysis, drafting and revision of the manuscript. MKT: Design of experiment, data analysis, critical manuscript writing and revision. ND: Provision of extra *in-vitro* assembly, technical support and manuscript reading. All authors read and approved the final manuscript.
